# Case Report: Surgical Intervention Under Pheochromocytoma Multisystem Crisis: Timing and Approach

**DOI:** 10.3389/fonc.2022.908039

**Published:** 2022-06-20

**Authors:** Shengjun Luo, Qingao Cui, Delin Wang

**Affiliations:** Urology Department, The First Affiliated Hospital of Chongqing Medical University, Chongqing, China

**Keywords:** pheochromocytoma multisystem crisis, VA-ECMO, emergency surgery, laparoscopy, pheochromocytoma

## Abstract

**Background:**

Progressive multiple organ failures still occur in some patients with pheochromocytoma multisystem crisis (PMC) despite α- and β-blockade being used, and emergency adrenalectomy may lead to rapid hemodynamic stabilization and recovery. Therefore, the optimal timing and surgical approach under PMC remain controversial.

**Case Presentation:**

A 50-year-old man presented with persistent chest pain accompanied by vomiting and headache. CT showed a right adrenal mass, and plasma catecholamine levels were significantly elevated. Phenoxybenzamine was used, but his symptoms were aggravated. He progressed to acute respiratory distress syndrome (ARDS) and received mechanical ventilation. Reexamination of CT showed pheochromocytoma rupture. Emergency pheochromocytoma resection was performed on the 5th day, and he was discharged on the 21st day. A 46-year-old woman was admitted for intrauterine device removal and received hysteroscopy under intravenous anesthesia. She presented with dyspnea, fluctuating blood pressure, and loss of consciousness 9 h after hysteroscopy surgery. CT showed a left adrenal mass, and plasma catecholamine levels were significantly elevated. Her condition fluctuated and could not meet the preoperative preparation criteria for pheochromocytoma despite adequate doses of α-blockade and β-blockade were taken. Furthermore, her lung condition worsened due to recurrent crises and pulmonary edema. After multidisciplinary discussions, laparoscopic left adrenalectomy with venoarterial extracorporeal membrane oxygenation (VA-ECMO) support was performed on the 28th day, and she was discharged on the 69th day.

**Conclusion:**

Elective surgical resection is the essential therapy for PMC with adequate preoperative medical management. Emergency surgery is recommended for patients who fail to achieve medical stabilization or progressive organ dysfunction within 1 week, especially those with tumor rupture and uncontrolled bleeding. The laparoscopic approach may represent an option even under PMC.

## Introduction

Pheochromocytoma multisystem crisis (PMC) is a rare, life-threatening condition inducing hemodynamic instability and multiple organ failures caused by the excessive release of catecholamine (1–3). The management of pheochromocytoma crisis includes initial medical stabilization, followed by appropriate α-blockade and fluid resuscitation before surgery (4, 5). However, progressive multiple organ failures still occur in some patients despite the use of α-blockade and β-blockade. Emergency adrenalectomy may lead to rapid hemodynamic stabilization and recovery, even though some are extracorporeal membrane oxygenation (ECMO)-assisted emergency adrenalectomy (2, 6, 7). However, the optimal timing of surgery under PMC remains controversial (2, 8). Minimally invasive surgery (laparoscopic or robotic) is considered the preferred surgical technique for small (<6 cm), non-invasive pheochromocytoma (1, 4); however, whether the laparoscopic approach suits patients with PMC also remains controversial (6). We present two cases combined with a literature review to determine the optimal timing and approach for surgery with unstable PMC.

## Case Presentation

### Patient 1

**Figure 1:**
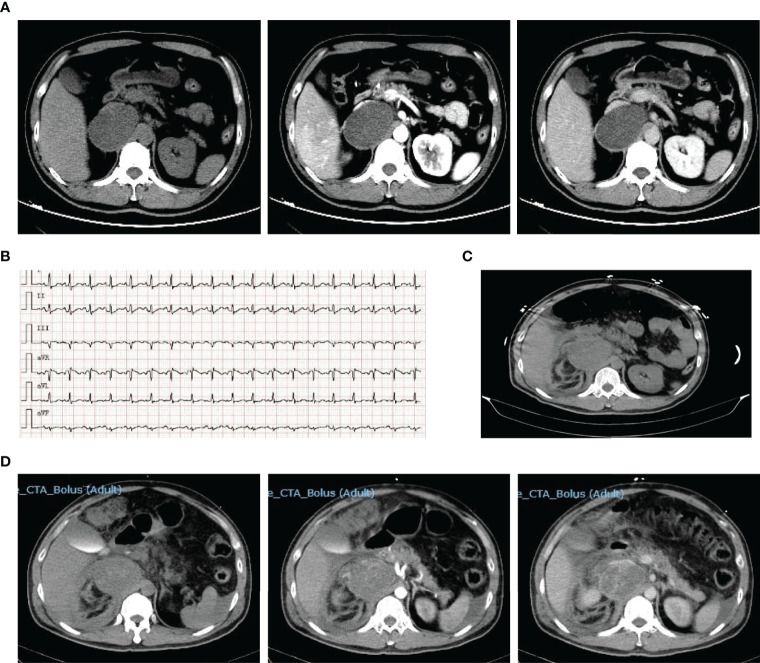
Representative images of patient 1. Contrast-enhanced CT on admission showed right adrenal mass **(A)**. ECG on the 3^rd^ day showed tachycardia **(B)**. CT scan on the 3^rd^ day showed pheochromocytoma rupture and hemorrhage **(C)**. Reexamination enhanced CT showed retroperitoneal hematoma on the 4^th^ day **(D)**.

A 50-year-old man presented with persistent chest pain accompanied by vomiting and headache for 5 h. He had a history of hypertension, which was well controlled by nifedipine, but he had not undergone a specialist examination to exclude secondary hypertension. His body temperature was 36.8°C, his blood pressure was 192/132 mmHg, his pulse rate was 95 bpm, and his respiratory rate was 20/min on admission. Serum cardiac markers showed 1.8 ng/ml of creatine kinase-MB (CK-MB), 287 ng/ml of myoglobin (MYO), 0.21 ng/ml of troponin (TNI), and 9.8 pg/ml of B-type natriuretic peptide (BNP). Routine blood tests showed that the total white blood cell count was 20.97 × 10^9^/L. Electrocardiography showed that the ST segment changed. The diagnosis was considered acute non-ST-segment elevation myocardial infarction, and emergency coronary angiography was performed; however, the coronary artery was not stenosis. Subsequent CT showed a 7.1 cm × 6.6 cm, round, right adrenal mass (**Figure 1A**). Plasma catecholamine metabolite levels (MNs) were significantly elevated (metanephrine, 573.5 ng/L; normetanephrine, 2,941.6 ng/L). Phenoxybenzamine (initial dose 10 mg, Q8 h) was used for preoperative preparation.

During hospitalization, the pain was aggravated and spread throughout the abdomen, accompanied by severe fluctuations in blood pressure and heart rate (**Figure 1B**). Furthermore, he progressed to acute respiratory distress syndrome (ARDS) and received tracheal intubation and mechanical ventilation on the 3rd day, and his hemoglobin decreased gradually from 159 g/L at admission to 75 g/L before surgery. CT reexamination showed pheochromocytoma rupture and hemorrhage (**Figure 1C**), and the retroperitoneal hematoma was increased after 1 day (**Figure 1D**). On the 5th day after admission, an emergency pheochromocytoma resection was performed with massive blood infusion. Pathological examination confirmed the diagnosis of pheochromocytoma rupture and hemorrhage. Plasma MN levels returned to normal ranges (metanephrine, 106.9 ng/L; normetanephrine, 80.5 ng/L). After 10 days of postoperative intensive care treatment and 6 days of general treatment, he was discharged on the 21st day of hospitalization. After discharge, his blood pressure returned to normal, and he did not need antihypertensive medication.

### Patient 2

**Figure 2:**
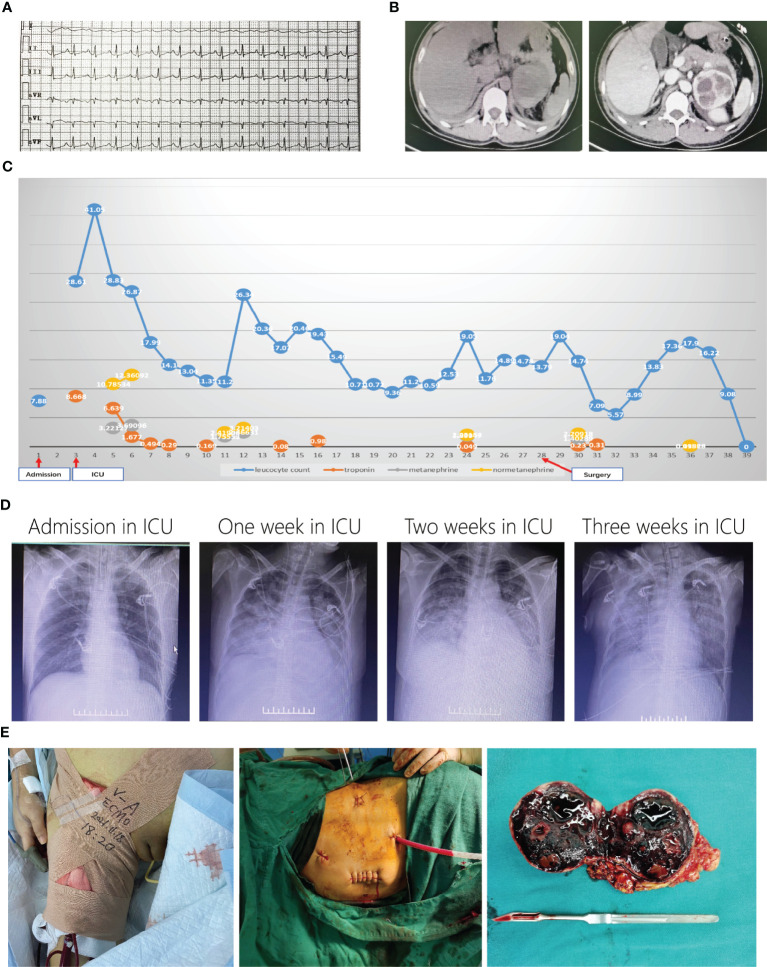
Representative images of patient 2. ECG showed tachycardia when admission in ICU **(A)**. Enhanced CT showed left right adrenal mass **(B)**. Curves of leukocyte count, troponin, metanephrine and normetanephrine showed recurrent crisis happened several times **(C)**. Chest X-ray changes after admission in ICU **(D)**. Laparoscopic surgery with ECMO support **(E)**.

A 46-year-old woman without a history of hypertension was admitted for removal of the intrauterine device and received a hysteroscopy under intravenous anesthesia. Transient ventricular tachycardia occurred during the operation. The patient experienced severe nausea and vomiting after surgery, which were considered side effects of anesthesia, until she presented with dyspnea, crackle, hypoxia, and fluctuating blood pressure and lost consciousness 9 h after hysteroscopy surgery. Her blood pressure fluctuated from 99/74 to 144/115 mmHg, and her pulse rate was 130–140 bpm (**Figure 2A**). Arterial blood gas analysis indicated severe acidosis (pH 7.0, 52 mmHg PO_2_, 51 mmHg PCO_2_). She received tracheal intubation and mechanical ventilation immediately. Large amounts of yellow fluid continued to drain out of her airway, and she was transferred to the intensive care unit (ICU). The total amount of leukocytes was significantly increased to 41.5 × 10^9^/L, procalcitonin was increased to 177.99 ng/ml, and plasma cardiac troponin was 8.668 ng/ml. During hospitalization in the ICU, her blood pressure fluctuated between 204/120 and 77/52 mmHg. Her condition was accompanied by coagulation disorders, renal failure, and liver dysfunction, and she received hemodialysis and blood transfusion. CT showed a 6.6 cm × 5.7 cm, round, central necrosis adrenal mass with an enhancing rim on the left side (**Figure 2B**).

PMC was considered, and plasma MN results (metanephrine, 3,221.27 ng/L; normetanephrine, 10,785.34 ng/L) confirmed the diagnosis. Phentolamine and esmolol were administered with continuous infusion, and blood volume expansion was performed. Her condition gradually stabilized. All indices improved significantly, and the tracheal catheter was removed on the 6th day after admission to the ICU (**Figure 2C**). However, her condition worsened 2 days later when her leukocyte count (26.34 × 10^9^/L) and plasma MNs (metanephrine, 2,466.31 ng/L; normetanephrine, 3,214.03 ng/L) significantly increased (**Figure 2C**). Her condition fluctuated, and she was not able to reach the preoperative preparation criteria for pheochromocytoma despite the administration of adequate doses of α-blockade and β-blockade. Furthermore, her lung condition worsened due to recurrent acute pulmonary edema and fibrosis (**Figure 2D**). After multidisciplinary discussions, an emergency left adrenalectomy with venoarterial extracorporeal membrane oxygenation (VA-ECMO) support was decided. Because of the trauma of open surgery and literature reports (6), we chose to use the laparoscopy approach first and prepared to open on the 28th day (**Figure 2E**). Pathology confirmed pheochromocytoma with hemorrhage, and plasma MN levels returned to normal ranges (metanephrine, 95.26 ng/L; normetanephrine, 118.99 ng/L). VA-ECMO was removed 6 days later, but mechanical ventilation lasted 20 more days after surgery. A long rehabilitation period was required due to pulmonary fibrosis. The patient was discharged on the 69th day for further pulmonary rehabilitation.

## Discussion

Pheochromocytomas and paragangliomas (PPGLs) are catecholamine-secreting neuroendocrine tumors (9). Excessive catecholamines released in a short period will cause a PPGL crisis, and the incidence ranges from 3% to 18% (1, 5, 10, 11). The clinical presentation of the PPGL crisis is widely variable and non-specific, and the classic triad, which consists of episodic headache, diaphoresis, and tachycardia, is only noted in some patients (10, 12). In our cases, patient 1 presented with chest pain, and patient 2 presented with nausea and vomiting as the initial clinical manifestations. Whitelaw divided the PPGL crisis into two types. Type A is described as a more limited crisis without sustained hypotension, whereas type B is described as a severe presentation with sustained hypotension, shock, and multiorgan dysfunction known as PMC (12).

PMC is a critical and lethal emergency, and organ-specific mechanical support is needed, including mechanical ventilation (85%), circulatory support (vasoactive drugs in 68% and ECMO in 41%), and renal replacement therapy (24%) (2, 7). Despite the use of the rescue methods noted above, the mortality of PMC remains high (15%–30%) (1, 7, 12).

Curative surgical resection is an essential treatment for PPGLs, and preoperative pharmacologic preparation (7–14 days) and fluid resuscitation are recommended (4, 5). Most PMC cases will be well controlled after internal medical treatment followed by sufficient α-blockade before surgery (5, 13). Emergency surgery is defined as taking inadequate α-blockade (time and dose) and failing to achieve medical stabilization before operation (5). However, in the case of life-threatening PMC with extreme hemodynamic fluctuations, emergency surgery may achieve hemodynamic stabilization rapidly (1, 2, 14, 15). In addition, surgery with ECMO support will improve the safety of the procedure (6, 7, 16). Delayed surgery may have a risk of recurrent crises, further organ failure, and more complications (7). In our report, patient 1 received emergency surgery due to tumor rupture and hemorrhage and recovered rapidly. Patient 2 experienced recurrent crises despite adequate α-blockade and β-blockade were used. Surgery was eventually performed under VA-ECMO but prolonged the hospitalization time and caused pulmonary fibrosis; thus, the recovery time was prolonged. Therefore, the optimal timing of surgery under life-threatening conditions remains controversial.

A previous cohort study and review of the literature suggested that emergency resection of pheochromocytoma should be avoided due to the high surgical mortality of 18% (5). However, most deaths occurred before 1990, and the mortality after 1990 was only 1 in 18 cases (6%). We performed a search of the English literature and identified 13 patients who presented with PMC and received emergency pheochromocytoma resection after 2010. All these patients were alive, including 4 who underwent surgery with the support of ECMO (1, 2, 6, 7, 14, 15). Furthermore, we extracted literature with specific data (**Table 1**). All cases were prepared by α-blockade for 1 to 20 days. Three patients underwent surgery within a week and were discharged earlier, whereas the other 3 patients who received longer preparation had longer hospitalization times and more complications. Therefore, the optimal timing of surgery is crucial. Emergency surgery enables rapid recovery, but with a high risk. Deferring surgery may cause the development of recurrent crises and further organ dysfunction. Because most cases will reach medical stabilization within 3–5 days after comprehensive treatment (5, 13), we recommend that emergency surgery should be performed if medical stabilization is not achieved or progressive organ dysfunction occurs within 1 week of treatment. In addition, tumor rupture and uncontrolled bleeding are strong indications for emergency surgery (5, 10).

**Table 1:** Emergency surgery under pheochromocytoma multisystem crisis in literature of recent years.

Author	Gender	Age (years)	Crisis manifestations				Tumor size (cm)	Duration of α-blockade before surgery (days)	Time from crisis to surgery (days)	Surgery approach	ECMO support	Complications	Duration of ICU stay (days)	Duration of hospital stay (days)	Outcome
			Severe hypertension/hypotension	Cardiac crisis	Pulmonary crisis	Other organ crisis									
Bekelaar T 2021 (2)	M	49	Yes	Yes	No	Renal, liver	Left, 5.7	1	1	Open	No	No	16	24	Alive
Choudhary M 2021 (6)	M	30	No	Yes	Yes	Renal	Right, 6.9	10	37	Laparoscopic	Yes	Reoperation of bleeding	N/A	75	Alive
UCHIDA N 2010 (15)	F	52	Yes	Yes	Yes	Renal	Left, 6	N/A	11	Open	No	N/A	N/A	106	Alive
Kakoki K 2015 (14)	M	70	No	Yes	Yes	Renal, liver	Left, 12	5	5	Open	No	N/A	N/A	42	Alive
Present case 1, 2022	M	50	No	Yes	Yes	Bleeding	Right, 7.1	3	5	Open	No	No	13	21	Alive
Present case 2, 2022	F	46	No	Yes	Yes	Renal, liver	Left, 6.6	20	26	Laparoscopic	Yes	Pulmonary fibrosis	46	69	Alive

ECMO, extracorporeal membrane oxygenation; ICU, intensive care unit; N/A, not applicable.

Minimally invasive surgery (laparoscopic or robotic) is the preferred approach for patients with pheochromocytomas (diameter <6 cm), even in patients presenting with pheochromocytoma crisis after medical stabilization (5, 17). However, the choice of a surgical approach varies according to the adrenal mass presentation, patient fitness for surgery, type and size of the tumor, surgeon’s experience, and hospital resources (17, 18). Therefore, a tumor size over 6 cm is not a contraindication for minimally invasive surgery. Choudhary reported that emergency laparoscopic surgery could also be performed on patients with uncontrolled PMC with the support of ECMO, despite the patient receiving secondary surgery for bleeding (6). Our case also showed that the laparoscopic approach might be an optional choice in unstable patients with PMC; however, open surgery should be considered the first choice for ruptured tumors (18), like in patient 1. The indications of surgical approaches during PMC are still not clear, mostly depending on the surgeon’s experience and multidisciplinary cooperation.

We presented 2 different cases with PMC. Patient 1 received emergency surgery due to intraperitoneal bleeding and recovered rapidly. Patient 2 experienced recurrent crises during preparation for surgery. Emergency surgery was eventually performed, but prolonged hospitalization and rehabilitation were required. Emergency surgery is recommended for patients with uncontrolled extreme hemodynamic fluctuations, progressive organ dysfunction, and tumor bleeding.

## Conclusion

PMC is a rare condition with variable manifestations, and elective surgical resection is an essential therapy with adequate preoperative medical management and involves the use of alpha-blocking agents. Emergency surgery is recommended for patients who fail to achieve medical stabilization or progressive organ dysfunction within 1 week, especially for patients with tumor rupture and uncontrolled bleeding. The laparoscopic approach may represent an option even under PMC. Due to the rarity of PMC, a large, randomized, and multicenter trial is needed to identify the best treatment and the optimal timing for surgery.

## Data Availability Statement

The raw data supporting the conclusions of this article will be made available by the authors, without undue reservation.

## Ethics Statement

The studies involving human participants were reviewed and approved by the Ethics Committee of the First Affiliated Hospital of Chongqing Medical University. The patients/participants provided their written informed consent to participate in this study. Written informed consent was obtained from the participant(s) for the publication of this case report.

## Author Contributions

DW and SL contributed to the conception and design of the study. SL and QC collected the data. SL and DW wrote the manuscript. All authors contributed to manuscript revision and read and approved the submitted version

## Conflict of Interest

The authors declare that the research was conducted in the absence of any commercial or financial relationships that could be construed as a potential conflict of interest.

## Publisher’s Note

All claims expressed in this article are solely those of the authors and do not necessarily represent those of their affiliated organizations, or those of the publisher, the editors and the reviewers. Any product that may be evaluated in this article, or claim that may be made by its manufacturer, is not guaranteed or endorsed by the publisher.
